# Gabapentin reverses central hypersensitivity and suppresses medial prefrontal cortical glucose metabolism in rats with neuropathic pain

**DOI:** 10.1186/1744-8069-10-63

**Published:** 2014-09-25

**Authors:** Hsiao-Chun Lin, Yu-Hsin Huang, Tzu-Hao Harry Chao, Wen-Ying Lin, Wei-Zen Sun, Chen-Tung Yen

**Affiliations:** Department of Life Science, National Taiwan University, No 1, Section 4, Roosevelt Road, Taipei, 10617 Taiwan; Department of Anesthesiology, National Taiwan University Hospital, Taipei, 10002 Taiwan

**Keywords:** Gabapentin, Allodynia, FDG-PET, Functional connectivity, Neuropathic pain

## Abstract

**Background:**

Gabapentin (GBP) is known to suppress neuropathic hypersensitivity of primary afferents and the spinal cord dorsal horn. However, its supra-spinal action sites are unclear. We identify the brain regions where GBP changes the brain glucose metabolic rate at the effective dose that alleviates mechanical allodynia using ^18^ F-fluorodeoxyglucose-positron emission tomography (FDG-PET) scanning.

**Results:**

Comparing the PET imaging data before and after the GBP treatment, the spared nerve injury-induced increases of glucose metabolism in the thalamus and cerebellar vermis were reversed, and a significant decrease occurred in glucose metabolism in the medial prefrontal cortex (mPFC), including the anterior cingulate cortex. GBP treatment also reversed post-SNI connectivity increases between limbic cortices and thalamus.

**Conclusions:**

Our results indicate that GBP analgesic effect may be mediated by reversing central hypersensitivity, and suppressing mPFC, a crucial part of the cortical representation of pain, in the brain.

## Background

Neuropathic pain results from damage or disease affecting the somatosensory system [1]. The clinical features of neuropathic pain include spontaneous pain, allodynia, and hyperalgesia [2]. Gabapentin (GBP), a voltage-dependent calcium channel α_2_δ subunit ligand, can effectively relieve neuropathic pain caused by painful diabetic neuropathy, postherpetic neuralgia, spinal cord injury, and phantom limb pain [3–6]. Previous studies have indicated that the analgesic effect of GBP acts primarily on the central nervous system, particularly at the supraspinal level [7, 8]. Reports have indicated that GBP binds to various brain regions [9, 10]; however, which brain areas are involved in the analgesic effect of GBP remains unclear.

Modern brain imaging techniques, such as functional magnetic resonance imaging (fMRI) and positron emission tomography (PET), are crucial for studying pain function [11–13], and can be used for screening effective analgesics [14–17]. Blood oxygenation level-dependent (BOLD) MRI provides a higher resolution rodent brain image; however, the changes of BOLD-MRI signals may not reflect the analgesic effect because the rodents were anesthetized. Instead, PET could provide the functional brain imaging of behaving animals. Although rodents must be anesthetized in the scanner for image acquisition, during the radiotracer uptake period, the rodents could be kept awake and responsive to appropriate stimulation [18–25]. For example, ^18^ F-fluorodeoxyglucose (FDG), a glucose analog that serves as a functional marker of glucose metabolism, requires 30 to 60 min for radiotracer distribution and uptake through circulation after injection into the tail vein of the rodent. During this uptake time the rodents can be subjected to various behavioral tests. Thus, FDG-PET could provide correlation between behavior and glucose metabolism in the rodent brain.

Recently, Kim et al. [26] and Thompson et al. [25] reported that regional metabolic changes in the brain of rat models of neuropathic pain could be revealed by the FDG-PET scanning. But they focused on the persistent, spontaneous pain after peripheral nerve injury. Allodynia, a painful sensation induced by an innocuous stimulus, is a major symptom of neuropathic pain [2]. It is unclear how mechanical allodynia affect the regional metabolic activity in the rat brain. To realize which brain areas would be altered under mechanical allodynia, and to reveal how the GBP exerts its analgesic effect in the brain, we used FDG-PET to investigate the glucose metabolic changes of the brain before and after GBP treatment under the mechanical allodynia state of neuropathic rats. We hypothesized that GBP works through the neuronal activity of the brain to exert its analgesic effect, and glucose metabolic changes could map such changes in neuronal activity.

## Results

### Mechanical allodynia developed after spared nerve injury (SNI) of sciatic nerve

**Figure 1:**
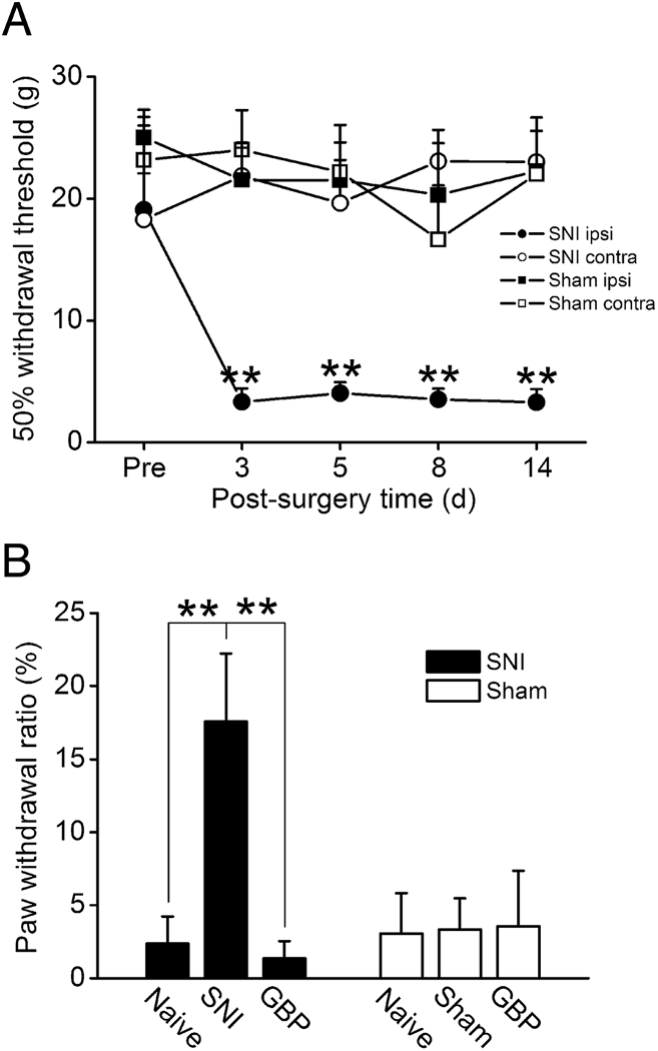
**Mechanical hypersensitivity after SNI and its reversal by GBP treatment. (A)** Mechanical allodynia was tested by von Frey filaments. The 50% withdrawal threshold of the ipsilateral hind paw of rats in the SNI group (n = 12) significantly decreased 3 d after surgery, and remained lower for at least 14 d. The withdrawal threshold of the contralateral hind paw of the SNI group and the bilateral hind paws of the sham group (n = 10) showed no significant changes after surgery. **indicates P < 0.01. Pre: pre-surgery. **(B)** Paw withdrawal ratio of the 6 g von Frey filament stimulation before the PET scan. In the SNI group (n = 12), the paw withdrawal ratio increased significantly after SNI surgery, and decreased significantly after GBP injection. The sham group (n = 10) showed no significant change in the paw withdrawal ratio. **indicates P < 0.01. Naïve: pre-surgery; SNI/Sham: post-surgery; GBP: post-surgery rats that received a gabapentin injection.

To verify whether the rats displayed mechanical allodynia after SNI surgery, we assessed the paw withdrawal responses using the von Frey filaments test (Figure 1). In the SNI group, the withdrawal threshold of the ipsilateral hind paw decreased significantly at 3 d after surgery, and maintained at least 14 d after surgery. The contralateral hind paw showed no change in the withdrawal threshold compared to its baseline. In the sham group, the withdrawal threshold of both bilateral hind paws did not change compared to their baseline. Thus the SNI rats developed mechanical allodynia after surgery. These behavioral test results also indicated that SNI rats showed consistency of behavioral hypersensitivity throughout the experimental course of PET scans.

### GBP alleviated mechanical allodynia

We tested whether GBP relieved mechanical allodynia in the neuropathic rats. Figure 1B shows the behavioral results before PET scanning. According to mechanical allodynia tests (Figure 1A), the normal (pre-surgery) paw withdrawal thresholds of rats were between 18 and 25 g. Thus we used the 6 g von Frey filament as an innocuous tactile stimulus. In the SNI group, at the first PET scan course, naïve rats that received a saline injection 60 min before stimulation rarely showed paw withdrawal responses (baseline ratio: 2.4 ± 1.9%) in the stimulation period. At the second PET scan course, the SNI-induced neuropathic rats that received a saline injection showed frequent paw withdrawal responses (17.6 ± 4.7%, *P* < 0.01 compared to the baseline ratio) in the stimulation period. At the third PET scan course, after GBP injection, the paw withdrawal responses of neuropathic rats decreased to the baseline level (1.4 ± 1.1%). GBP significantly alleviated the paw withdrawal ratio (*P* < 0.01, compared to the ratio of post-surgery stimulation) in the SNI rats. Rats in the sham group did not show apparent paw withdrawal behavior, and GBP given to these rats did not alter this.

### Brain glucose metabolism mapping in response to GBP treatment

**Figure 2:**
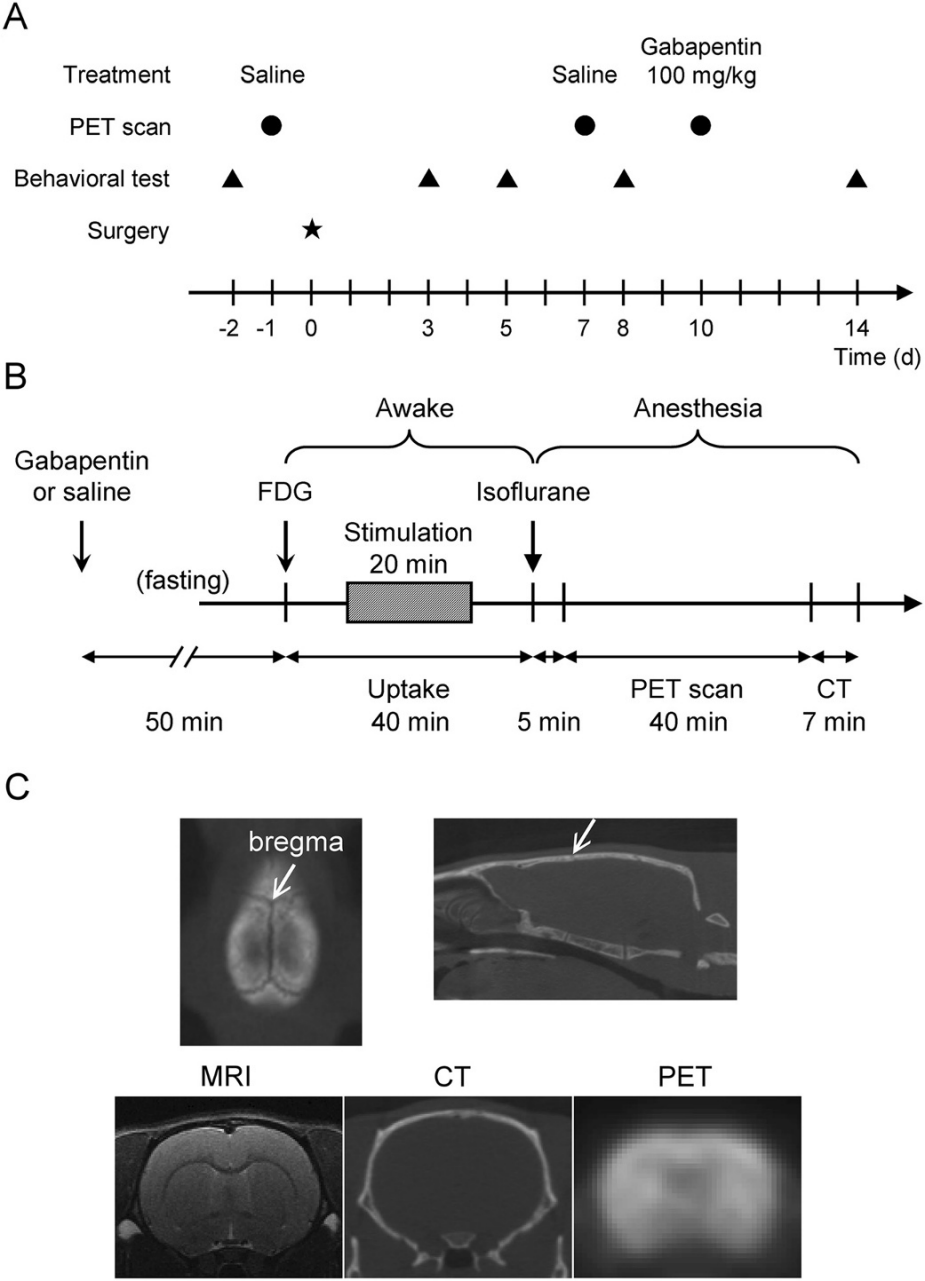
**Experiment design and image co-registration landmarks. (A)** Time course of the study. **(B)** The FDG-PET scans procedure. **(C)** Upper row shows the bregma (arrow) in the CT images. The CT images scanned in the same session were used to align the PET images. Horizontal level line was determined first as in the right panel, and the bregma was determined in the horizontal plan (left panel). PET and CT images were resliced accordingly. Lower row shows resliced coronal images at the level of bregma. Note anterior commissure in the MRI image was used to match the level of bregma.

To compare the changes of glucose metabolic activity in the brain of neuropathic rats before and after GBP treatment, each individual rat was scanned 3 times, first before and the second time 7 days after sciatic nerve injury, and the third time after GBP treatment (Figure 2A and B). FDG uptake values were normalized, and data from different rats aligned according to bregma and anterior commissure (Figure 2C).

**Figure 3:**
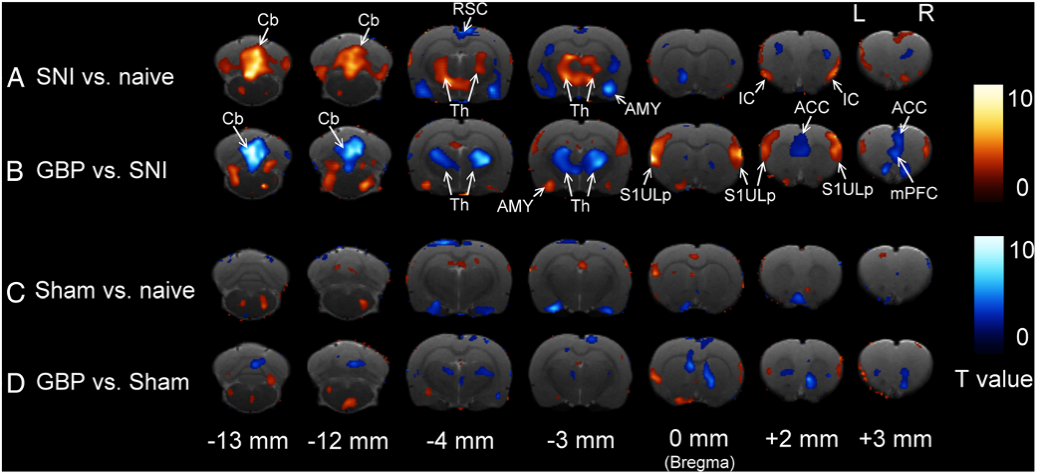
**GBP action sites in the brain.** Statistical t-map of PET images showed significant increases (warm color) and decreases (cold color) of glucose uptake in the brains of SNI (upper rows) or sham (lower rows) rats between the first and second scan, **(A)** SNI vs. naïve and **(C)** Sham vs. naïve, or the second and third scan, **(B)** GBP vs. SNI and **(D)** GBP vs. Sham. The PET scans are superimposed on the coronal sections of the brain. L and R indicate the left (ipsilateral) and right (contralateral) brain, respectively. SNI group, n = 12; Sham group, n = 10. Abbreviations please refer to the text.

Figure 3 shows the statistical parametric mapping (SPM) results. In the SNI group, comparing the SNI to the naïve condition (Figure 3A), the glucose metabolism increased in the bilateral anterior insular cortex (AIC, +2 mm), thalamus (-3, -4 mm), and cerebellar (Cb) vermis (-12, -13 mm), whereas it decreased in the contralateral amygdala (AMY, -3 mm) and bilateral retrosplenial cortex (RSC, -4 mm). Comparing the SNI rats that received the GBP treatment to the SNI condition (Figure 3B), the glucose metabolism decreased in the mPFC (+3 mm), anterior cingulate cortex (ACC, +2, +3 mm), thalamus (-3, -4 mm), and Cb vermis (-12, -13 mm), whereas it increased in the bilateral upper lip regions of primary somatosensory cortex (S1ULp, +2 to 0 mm) and ipsilateral AG (-3 mm). In the sham group, comparison between before and after sham-surgery only showed slight alteration of glucose metabolism, such as ipsilateral S1ULp (0 mm, Figure 3C). The sham group that received GBP injection (Figure 3D) showed slight decrease of glucose metabolism in the basal ganglia (0 mm), and increased relative small area in the ipsilateral S1ULp (0 mm).

### Region of interest (ROI) analysis verified the SPM maps

To verify the SPM results, we quantified the relative activity changes of specific brain structures as activation index (AI) values. Table 1 shows the results of the ROIs analysis. In the SNI group, comparing the SNI to the naïve condition, the AI increased significantly in the bilateral IC, the ipsilateral posterior thalamic nuclei (Po) and ventral posterior thalamic nuclei (VP), and Cb vermis, whereas it decreased significantly in the contralateral AMY and bilateral RSC. Comparing the GBP to the SNI condition, the AI decreased significantly in the bilateral mPFC, the ipsilateral ACC, the bilateral Po, the contralateral VP, and the Cb vermis, whereas it increased significantly in the bilateral S1ULp, barrel field region of S1 (S1BF) and secondary somatosensory cortex (S2), and the ipsilateral AMY. Although the SPM maps displayed slight changes of glucose metabolism, the sham group showed no significant changes of AI in the cortex, thalamus, and cerebellum compared to the naïve condition or the sham rats that received a GBP injection. According to the ROI analysis, these results were consistent with the SPM imaging maps.

**Table 1:** **Activation index (%) by region of interest (ROI) analysis in different conditions of SNI and sham groups**

ROIs	SNI group (n = 12)			Sham group (n = 10)		
	Naïve	SNI	GBP	Naïve	Sham	GBP
mPFC i	36.5 ± 2.1	35.2 ± 2.0	* 29.6 ± 1.1	34.6 ± 2.2	35.9 ± 1.3	31.5 ± 1.6
mPFC c	37.9 ± 1.8	35.0 ± 2.0	* 29.7 ± 1.2	33.1 ± 2.2	33.5 ± 1.5	28.6 ± 2.0
ACC i	36.9 ± 1.7	37.4 ± 2.6	* 31.4 ± 1.3	37.3 ± 1.8	37.9 ± 1.5	34.1 ± 2.8
ACC c	34.7 ± 1.4	35.6 ± 2.6	32.3 ± 1.4	34.5 ± 1.8	35.8 ± 1.9	32.2 ± 2.8
RSC i	20.0 ± 1.7	# 14.8 ± 2.4	14.5 ± 2.1	18.9 ± 1.7	15.1 ± 2.3	15.4 ± 2.4
RSC c	20.2 ± 1.6	# 14.8 ± 2.3	13.7 ± 2.2	18.8 ± 1.9	15.0 ± 2.8	15.0 ± 2.8
IC i	1.4 ± 1.5	# 4.8 ± 1.4	3.6 ± 1.8	1.3 ± 2.1	0.3 ± 2.1	1.6 ± 3.1
IC c	1.3 ± 1.4	# 5.1 ± 1.5	3.6 ± 1.6	1.4 ± 2.0	0.5 ± 2.1	1.5 ± 3.0
S1HL i	14.7 ± 1.3	15.7 ± 1.3	15.1 ± 2.0	15.3 ± 1.6	12.3 ± 1.5	13.0 ± 3.1
S1HL c	15.3 ± 1.6	15.7 ± 1.6	15.2 ± 1.1	13.5 ± 2.3	12.5 ± 1.6	13.0 ± 3.0
S1BF i	18.7 ± 0.9	18.0 ± 1.6	** 25.0 ± 1.0	18.5 ± 1.3	18.5 ± 1.1	21.5 ± 2.4
S1BF c	18.6 ± 1.0	18.7 ± 1.5	** 25.7 ± 1.0	18.5 ± 1.3	17.6 ± 0.8	22.6 ± 2.4
S1ULp i	19.4 ± 1.6	19.7 ± 1.5	*** 27.2 ± 1.2	21.8 ± 1.3	22.6 ± 1.8	25.9 ± 2.0
S1ULp c	17.9 ± 1.5	19.3 ± 1.6	** 27.1 ± 1.4	22.3 ± 2.0	21.1 ± 0.9	24.0 ± 2.2
S2	9.8 ± 1.9	10.4 ± 1.7	* 16.4 ± 1.9	11.9 ± 1.4	10.4 ± 1.8	15.2 ± 2.0
S2	10.4 ± 2.2	10.8 ± 1.8	** 20.1 ± 1.4	14.3 ± 1.7	10.8 ± 1.3	13.6 ± 2.1
M1 i	14.1 ± 2.3	15.2 ± 1.5	15.5 ± 0.8	16.2 ± 1.8	15.3 ± 2.0	16.7 ± 3.1
M1 c	13.9 ± 2.9	13.3 ± 1.8	16.1 ± 1.1	14.6 ± 1.7	15.3 ± 2.1	15.2 ± 2.8
CPu i	31.8 ± 1.6	28.6 ± 1.9	28.6 ± 1.3	28.6 ± 1.5	28.9 ± 1.4	28.7 ± 2.2
CPu c	33.7 ± 1.2	28.8 ± 1.9	29.7 ± 1.4	30.4 ± 1.4	28.9 ± 1.5	29.5 ± 2.2
Po i	21.4 ± 1.1	# 26.2 ± 1.6	** 21.0 ± 1.3	22.2 ± 1.2	23.9 ± 1.2	21.8 ± 2.3
Po c	24.5 ± 1.0	27.8 ± 1.7	** 22.3 ± 1.4	23.7 ± 1.2	25.0 ± 1.4	23.3 ± 2.1
VP i	8.4 ± 1.1	## 13.2 ± 1.2	9.5 ± 1.1	11.1 ± 1.3	10.0 ± 1.2	8.2 ± 1.8
VP c	9.3 ± 1.0	12.3 ± 0.9	* 9.2 ± 1.1	10.1 ± 1.3	11.0 ± 1.2	8.5 ± 1.7
AMY i	-9.3 ± 1.2	-11.3 ± 1.3	* -7.9 ± 1.2	-10.9 ± 0.9	-13.0 ± 1.4	-10.4 ± 0.9
AMY c	-5.3 ± 1.2	# -7.0 ± 1.1	-5.8 ± 1.4	-8.1 ± 0.6	-10.0 ± 1.3	-9.3 ± 1.0
Cb vermis	4.7 ± 1.7	## 12.0 ± 1.2	** 6.9 ± 1.2	5.2 ± 1.7	5.6 ± 1.7	7.2 ± 2.0

Post-surgery (SNI) compared to pre-surgery (Naïve) by the one-way repeated measures ANOVA, #*P* < 0.05, ##*P* < 0.01. Gabapentin treatment (GBP) compared to post-surgery by the one-way repeated measures ANOVA, *P < 0.05, **P < 0.01, ***P < 0.001. i: ipsilateral; c: contralateral. Data were presented as mean ± SEM. Abbreviations refer to the main text.

### Functional connectivity of the brain regions in different conditions

**Figure 4:**
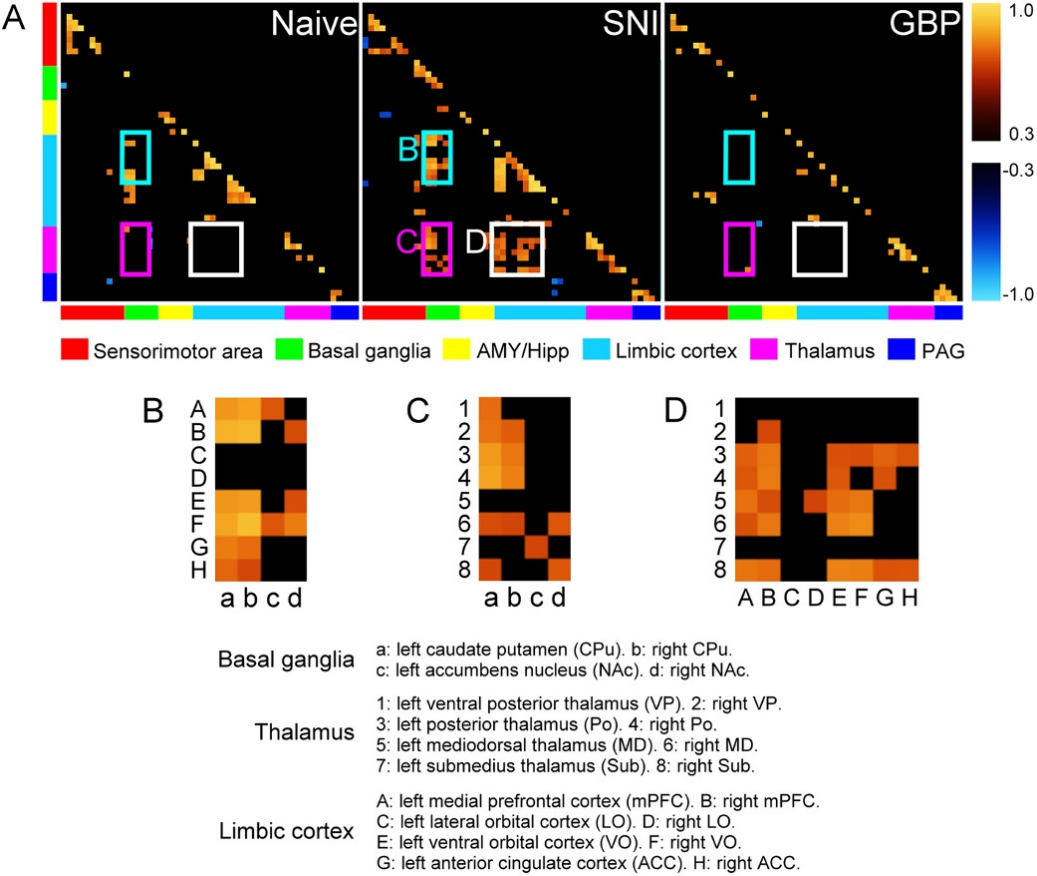
**Functional connectivity in the brain of pre-SNI, post-SNI and GBP-treated conditions. (A)** Interregional correlation matrices of the 3 conditions of SNI rats. Pearson’s correlation coefficients were color-coded. The warm and cold color scales stand for positive and negative significant correlations, respectively. Note the strong positive connections were appeared in the **(B)** basal ganglia to limbic cortex, **(C)** basal ganglia to thalamus, and **(D)** thalamus to limbic cortex. The enlarged rectangles in **(B)** to **(D)** represent the detail of correlation matrices boxed in A. SNI rats, n = 12.

To reveal the correlations of glucose metabolism among distinct brain regions in different conditions, we classified six functional groups and displayed the cross-correlation matrices showing the functional connectivity of SNI rats (Figure 4). Before SNI surgery (the naïve state), the distinct brain regions showed sparse correlation. After SNI surgery, apparent correlations were represented among various brain regions. There were 3 main clusters appeared in the interregional correlation matrix, including basal ganglia to limbic cortex, basal ganglia to thalamus, and thalamus to limbic cortex. The basal ganglia showed more connectivity with thalamus and limbic cortex after SNI surgery, especially in the bilateral caudate-putamen (CPu, Figure 4B,C). We further focused in particular on the functional connectivity between thalamus and limbic cortex (Figure 4D). The selected ROIs of thalamus included bilateral VP, Po, MD, and submedius thalamic nucleus (Sub). The selected ROIs of limbic cortex included bilateral mPFC, ACC, lateral orbital cortex (LO), and ventral orbital cortex (VO). After SNI surgery, the mPFC showed more connectivity with selected thalamic nuclei except ipsilateral VP and Sub. The VO showed well connectivity with thalamus except bilateral VP and ipsilateral Sub. The ACC showed less connectivity with bilateral Po and contralateral Sub. The SNI-induced correlations were nearly reversed after GBP treatment. In general, the two functional groups displayed notable correlation after SNI surgery, and GBP disputed this connectivity.

## Discussion

By serial longitudinal FDG-PET scanning of the same rat before, during neuropathic states and immediately following GBP treatment, we obtained 3 major findings in the present study. (1) Glucose metabolism increased in the bilateral IC, thalamus, and Cb vermis, and decreased in the AMY and bilateral RSC in SNI rats under mechanical allodynic stimulation. (2) GBP treatment reversed malfunction of thalamus, AMY and Cb vermis in the SNI rats, and suppressed the glucose metabolism in the mPFC. And (3) functional connectivity between thalamus and limbic cortical structures increased in SNI rats. This increase was alleviated by GBP treatment. Although GBP-induced pharmacological effects in the rat brain have been revealed by pharmaco-MRI (phMRI) or fMRI [14–16, 27] studies, to our knowledge, this study is the first PET report to demonstrate the action of GBP in the brain of neuropathic rats.

Allodynia is a major symptom in neuropathic pain patients, and its brain mechanism remains an open issue. Several studies focused on the functional abnormalities in the brain of allodynic state of the patients with neuropathic pain [28–32]. Brain areas including S1, secondary somatosensory cortex (S2), ACC, IC, mPFC, and thalamus [11, 33] have been found changed activity. Because the difficulty in patient recruitment, different etiology and disease history might alter the brain areas in these studies. It is also ethically difficult to follow the same human patient longitudinally before and during the development of neuropathic pain. In the present study, we used animal model as a surrogate to probe the functional changes in the brain of SNI rats. We performed an innocuous mechanical stimulation during the FDG uptake period while the rats were awake in the testing apparatus. The behavioral results indicated that rats after SNI surgery exhibited frequent paw withdrawal behavior, which suggested the neuropathic rats might experience tactile allodynia. In our observations, the neuropathic rats in response to the light tactile stimulation not only withdrew their paw, but also frequently accompanied with flinching or licking of the paw, suggesting these behaviors were not merely the simple reflex to the stimulation. When GBP was injected to the neuropathic rats, these abnormal behaviors were alleviated significantly.

Recently, Kim et al. [26] and Thompson et al. [25] reported the changes of glucose metabolic activities in the brain of neuropathic rats under the resting state. The alterations they observed could implicate the functional adaptation of spontaneous pain in the rat brain. In the present study, the PET scan results which compared the changes of glucose metabolic activities before and after SNI surgery revealed that IC, thalamus, and Cb vermis showed hyper-activation, whereas AMY and RSC showed hypo-activation. These metabolic activity changes might be the signatures of the mechanical allodynia in the rat brain. In the cortical level, anterior IC plays a major role in the cortical representation of pain and is part of the affective-motivational components of pain [34]. IC is considered as an integration region of sensory and emotional inputs. Several PET studies showed IC activation of neural activity in the allodynia state [29, 32, 35]. Our results suggested that bilateral IC activation related to the allodynia state of neuropathic rats. The RSC showed decreased glucose metabolism in the neuropathic rats. Although the functions of RSC in nociception processing remain to be elucidated, it was suggested the alteration of RSC might be relate to change of default mode network [36, 37].

In the subcortical level, the activation of the thalamus might relate to the nociceptive response. Previous studies indicated that damage to the somatosensory system caused higher neuronal activity [38, 39] and glucose utilization [40] in the thalamus. The ROI analysis further revealed significant activation in the ipsilateral PO and VP, but not in the contralateral side. Iadarola et al. [41] reported that patients with chronic neuropathic pain displayed deactivation of contralateral thalamic activity under PET scanning. It was expected that painful stimuli would induce activation of thalamic activity in the contralateral side. According to the ROI analysis, after spared nerve injury, the bilateral thalamus (including Po and VP) showed increased glucose metabolism. There was no statistical significance between the ipsilateral and contralateral thalamus. The AMY showed deactivation in our study. The AMY is involved in several aspects of affective information processing, including pain [42]. Several functional imaging studies have reported the decreased activity in AMY during painful stimulation [28, 43, 44]. Although our results indicated that contralateral AMY showed significant deactivation, according to ROI analysis (Table 1), there was a trend that bilateral AMY decreased glucose metabolism under allodynic state. In addition to the forebrain, the activation of the cerebellum was also observed. The activation of the cerebellum during allodynic stimulation might not only relate to paw withdrawal behavior, but play a role in sensory processing [45].

One might argue that there was no activation in the contralateral hindlimb region of S1 (S1HL) in our study. Neither SPM results nor ROI analysis showed elevated glucose metabolic activity in the bilateral S1HL. SI activation was not found in a recent FDG-PET study of the spinal nerve ligated rat [26]. The activation of S1 in the neuropathic pain patients is also controversial [46]. Several studies reported that brush-evoked allodynia in neuropathic patients increased regional cerebral blood flow (rCBF) or BOLD signals in the S1 [28–30]. However, using similar experimental design to detect rCBF in the neuropathic patients during brush-evoked allodynia, Witting et al. [32] reported that there were no significant changes in the thalamus, S1, and ACC. The absence of S1 activity might be the pathophysiological consequence of the animal model we used. Even though our results indicated that thalamus showed robust activation after allodynic stimulation. Seminowicz et al. [47] reported that after spinal cord injury, rats displayed functional disconnection between thalamus and S1. This finding provided another possible mechanism for the absence of S1 activity. We also performed functional connectivity analysis. Our data indicated that activation in the thalamus had higher correlation with limbic cortex but not with somatosensory cortex in the SNI rats.

After receiving GBP treatment, several brain regions reversed their metabolic activity patterns back to their baseline levels before nerve injury. The most prominent changes were present in the thalamus, AMY and Cb vermis. The thalamus showed activation under the allodynia state, and after GBP treatment, the increased glucose metabolism reverted to the baseline level (Table 1). The reversion of thalamic activity by GBP treatment was similar to Takemura et al. [27], though our methodology was different. The ROI analysis indicated a significant decrease in the bilateral PO and contralateral VP, which might reflect the analgesic effect of GBP. Deactivation also presented in the cerebellum. Because the paw withdrawal ratio reduced substantially, the decreased glucose metabolism might reflect reduced neural activity. Hooker et al. [15] demonstrated that the cerebellum showed activation of BOLD signals after GBP infusion. This discrepancy might be due to that their animals were anesthetized, whereas our animals were awake. Activation of the AMY might be another manifestation of reversed neural activity induced by GBP treatment.

Besides, the mPFC and ACC showed specific deactivation after GBP treatment. The function of mPFC is related to affect, emotion, memory and decision making [34]. Millecamps et al. [48] reported that directly injected a partial agonist of NMDA receptor into mPFC could induce antinociception in SNI rats, but not into thalamus or IC. Jiang et al. [49] described that antinociception by motor cortex stimulation would suppresses the BOLD signals in the mPFC. These studies suggested that mPFC play an important role in regulating pain processing in the brain. The ACC is one of the main cortical structures in regulating the affective and emotional component of pain [34]. Takemura et al. [27] described that after GBP injection, the increased BOLD signals of neuropathic rats would revert to baseline. Recent studies indicated that after nerve injury, the glutamate transmission augmented in the ACC [50–53]. Bak et al. [54, 55] demonstrated that glucose utilization is correlated with glutamatergic cell activity. Although mPFC and ACC did not change their glucose metabolism under the allodynic stimulation, after GBP treatment, the mPFC and ACC showed significant deactivation. This deactivation of neural activity is similar to GBP-induced pharmacodynamic effects in the neuropathic rats [15]. The sham group animals which received the same dosage of GBP injection did not alter the glucose metabolism in the mPFC and ACC. These neuropathic-specific GBP changes might relate to the analgesic effect of the drug. The S1ULp, S1BF and S2 showed strong activation after GBP treatment in the neuropathic rats. The glucose metabolism of the sham group showed a trend of increase in the S1ULp, S1BF and S2 after GBP treatment, although the changes did not reach statistical significance. Though we did not realize the mechanism of such finding, the relative high glucose metabolism of the S1ULp, S1BF and S2 might be a specific effect of the GBP treatment in the neuropathic rats.

This study included several limitations. First, FDG-PET cannot discriminate excitatory from inhibitory neural activity, because both neural activities consume energy and increase the glucose metabolic rate. Deactivation of neural activity might reflect reduced neural activity; however, it cannot differentiate whether the deactivation is caused by the suppressed glutamatergic cells or the inhibitory outcome of activation of GABAergic cells. Further electrophysiological experiments are needed to investigate whether the metabolic activity changes are neuronal. Second, our acquired PET images were static and cumulative signal counts of the total FDG uptake period, and thus contained no dynamic information. Third, the GBP was injected systemically, thus the changes of glucose metabolism in the brain might not be the direct effect of the drug. It cannot separate the spinal or supraspinal mechanisms of the drug effect, the functional brain changes only represent the total analgesic effect of the drug. Nevertheless, because of numerous binding sites of GBP [9, 10] and the preferential action [7, 8] in the brain, our finding might implicate the probable areas that involved in the analgesic effects of GBP.

## Conclusions

GBP is the first-line analgesic in relieving neuropathic pain. We used longitudinal FDG-PET to measure the change in brain metabolic activity during the analgesic action of GBP in awake neuropathic rats. Our findings suggest that mPFC, ACC, thalamus, and cerebellum might be the primary sites of analgesic action of GBP. By means of FDG-PET scan, we could recognize the action sites of analgesics in the brain of awake animals, and this methodology can serve as a promising tool for understand the effect of the analgesic drugs.

## Methods

### Animals

We used 22 male Sprague–Dawley rats in this study. Six-week-old rats (weighing from 180 to 210 g) were purchased from BioLasco Company in Taiwan. Groups of 2 to 3 rats were housed together in plastic cages and were placed in a temperature- and humidity-controlled room (23 ± 2 °C and 55 ± 5%) with a 12-h light/dark cycle (lights on at 06:00 h). Food and water were available ad libitum. All animal care and experimental procedures were approved by the Institutional Animal Care and Use Committee of National Taiwan University. The guidelines established by the Codes for Experimental Use of Animals from the Council of Agriculture of Taiwan, based on the Animal Protection Law of Taiwan, were adhered to.

### Surgery

We used the SNI model for neuropathic pain. The experimental surgery procedure was in accordance with Decosterd and Woolf [56]. Under sodium pentobarbital (50 mg/kg, i.p.) anesthesia, we exposed the sciatic nerve of the left leg and its trifurcate branches. We tightly ligated the common peroneal and tibial nerves with 6.0 silk and cut distally to the ligation site, leaving the sural nerve intact. After surgery, we closed the muscle and skin in 2 layers. For sham surgery, we exposed the sciatic nerve and its branches. The wound was closed in 2 layers without additional operation of the sciatic nerve branches.

### Drug and treatment regimen

GBP powder (purity 98%) was purchased from Tokyo Chemical Industry Co., Ltd., Japan. GBP powder was dissolved in 0.9% saline and freshly prepared for each experiment. We obtained the GBP dosage (100 mg/kg) used in this study from previous studies [57–59], and validated the efficacy of this dosage of GBP with preliminary tests. A bolus of GBP solution was injected intraperitoneally 60 min prior to behavioral stimulation for the drug to reach its maximum analgesic effect (Figure 2B) [58].

### Behavioral tests

To confirm whether the rats developed neuropathic pain after surgery, we assessed the mechanical allodynia using the von Frey filaments (North Coast Medical, Inc., Morgan Hill, USA) test. Each rat was individually placed on an elevated wire mesh floor in the transparent acrylic box (dimensions 21 cm × 12 cm × 14 cm) and allowed to acclimate for 10 min. We applied the von Frey filaments of various bending forces (0.6, 1, 2, 4, 6, 8, 15, and 26 g) to the lateral plantar surface, innervated by the sural nerve of the hind paw, to test the withdrawal response. The behavioral test procedure was in accordance with Chaplan et al. [60]. Brisk withdrawal or paw flinching was regarded as positive response. At the beginning, the 2 g von Frey filament was applied to the hindpaw, the next stronger or weaker filaments were applied according to the prior response (positive or negative). The response patterns and 50% withdrawal threshold were calculated based on Chaplan et al. [60]. To prevent the influence of the behavioral test from the mechanical stimulation of the PET scan course, we assessed the tactile withdrawal thresholds on the following days: 2 d before surgery as the baseline, and 3, 5, 8, and 14 d after surgery (Figure 2A).

### FDG-PET scan protocol

The time course of FDG-PET scans and scanning procedure are shown in the Figure 2A. The rats were randomly divided into SNI (n = 12) and sham (n = 10) groups. Each rat underwent the PET scan 3 times. The first scan (naïve control) was performed 1 d before surgery, the second scan (neuropathic/sham) 7 d, and the third scan (neuropathic/sham with gabapentin treatment) 10 d after surgery.

The rats were fasted overnight before each scan to enhance FDG utilization during the PET experiment. For PET study, each rat received 2–2.5 mCi of FDG by tail vein injection with the aid of brief (less than 2 min) isoflurane inhalation (5% in 100% oxygen). After FDG injection, the rat was placed immediately into a transparent acrylic box, which was constituted with wire mesh floor for von Frey filament to pass through. The rat was allowed 40 min for FDG uptake before the PET scanning. During the FDG uptake period, the first 10 minutes were allowed for the rat to recover from anesthesia. We used a 6 g von Frey filament to stimulate the lateral plantar surface of the left (nerve-injured) hind paw of the rat from 10 to 30 min after FDG injection. The left hind paw received mechanical stimulation once every 5 s during the 20 min stimulation period (a total of 240 stimulations). The duration of each stimulus was 1 s. We counted the paw withdrawal times and transformed the results as the withdrawal ratio (in percentage). After mechanical stimulation, each rat was allowed to rest for another 10 min. At the end of the 40 min FDG uptake period, the rat was anesthetized by isoflurane inhalation (5% in 100% oxygen). After reaching a deep state of anesthesia, the rat was placed into the PET scanner with the head holder (to prevent motion artifact) and continuously maintained with isoflurane inhalation (2% in 100% oxygen) until the end of the scan. The scan time was 40 min for PET and 7 min for computed tomography (CT).

The FDG uptake in the brain was measured using an eXplore Vista Dual-Ring Small-Animal PET/CT scanner (GE Healthcare, Waukesha, WI) with an average full width at half maximum (FWHM) resolution of 1.26 mm. The images were first anatomically standardized to achieve symmetrical midline alignment. To improve the resolution and sensitivity of acquired images, the images were reconstructed by the 3D ordered subsets expectation maximization (OSEM) algorithm. The nominal voxel size was 0.387 mm × 0.387 mm × 0.775 mm. Each of the 61 transverse slices in the reconstructed images contained 175 × 175 voxels. FDG uptake by brain regions was quantified as standardized uptake values (SUVs) using the formula:


### PET images processing

We performed the PET images processing according to our previously published voxel-based SPM analytical method [21]. Briefly, we used the software Medical Image Processing, Analysis and Visualization (MIPAV, version 4.1.2, Center for Information Technology, National Institutes of Health, USA) and SPM (SPM8, Wellcome Trust Centre for Neuroimaging, Institute of Neurology, UCL, United Kingdom) to do the image preprocessing and to generate the statistical parametric maps. For image preprocessing: (1) To facilitate images re-alignment, we rotated and translated each brain images (PET/CT/MRI) to a standard space, and adjusted the resolutions to the same attributes by MIPAV. The corresponding, size-matched MRI images were obtained from our lab’s MRI database, which served as anatomical images. The slice of bregma in CT images and the slice of anterior commissure in MRI images were chosen as indices of the coordinate origin to align these 2 sets of images. Because eXplore Vista PET/CT scanner provided the same coordinates of the PET and CT images, then PET images could be aligned with the MRI images (Figure 2C). (2) The raw PET images were first coregistered to their corresponding, size-matched T2-weighted MRI images. (3) For each PET image, the signals outside the brain were cropped with a brain mask; the brain mask was drawn manually from their corresponding, size-matched MRI image using MIPAV. (4) The cropped PET images were further coregistered, spatial normalized, and resliced to a template T2-weighted MRI image (coordinate corrected) [61], and the final voxel size of the preprocessed PET images was 0.2 mm × 0.2 mm × 0.2 mm. The statistical parametric maps were generated using the voxel-wise paired t test and 2-sample t test for intra-subject and inter-subject comparison, respectively. Global normalization scaling was applied. Significant clusters were determined according to an individual voxel threshold of *P* < 0.05 with at least 30 continuous voxels.

### ROI analysis

To verify the accuracy and consistency of results represented by SPM, we further investigate the relative activities of specific brain structures, which showed significant changes of glucose metabolism among different conditions, by ROI analysis. The specific brain structures included mPFC, ACC, RSC, IC, S1HL, S1ULp, S1BF, S2, primary motor cortex (M1), CPu, Po, VP, AMY, and Cb vermis. All bilateral structures except Cb vermis were separately analyzed. We used OsiriX Imaging software (Pixmeo, Geneva, Switzerland) to extract the SUVs of selected ROIs. We merged the PET images with the corresponding, size-matched MRI images by means of CT coordinates. All the ROIs were delineated with the aid of corresponding MRI images according to the atlas of the rat brain [62]. To quantify the individual ROI activity as a percentage difference of average total activity of the entire brain, we calculated the AI [63] as the formula:


### Connectivity analysis

To determine whether the correlations of glucose metabolism among distinct brain regions were altered before SNI, after SNI and after GBP treatment, we performed the functional connectivity analysis. Cross-correlation matrix of brain areas were calculated using inter-subject variability of brain FDG consumption. The ROIs of brain areas were defined according to the atlas of the rat brain [62]. The selected ROIs divided into six functional groups (Figure 4A): sensorimotor area, basal ganglia, AMY/hippocampus (Hipp), limbic cortex, thalamus and periaqueductal gray (PAG). The sensorimotor area group includes Cb vermis, left M1, right M1, left secondary motor cortex (M2), right M2, left forelimb region of S1 (S1FL), right S1FL, left S1HL, right S1HL, left S2, and right S2, respectively. The basal ganglia group includes left CPu, right CPu, left nucleus accumbens (NAc), right NAc, left ventral pallidum, and right ventral pallidum, respectively. The AMY/Hipp group includes left basolateral AMY (BLA), right BLA, left central nucleus of AMY (CeA), right CeA, left Hipp, and right Hipp, respectively. The limbic cortex groups include left mPFC, right mPFC, left lateral orbital cortex (LO), right LO, left ventral orbital cortex (VO), right VO, left ACC, right ACC, left middle cingulate cortex (MCC), right MCC, left RSC, right RSC, left posterior IC (PIC), right PIC, left AIC, and right AIC, respectively. The thalamus group includes left VP, right VP, left Po, right Po, left mediodorsal thalamic nucleus (MD), right MD, left Sub, and right Sub, respectively. The PAG group includes dorsal PAG, left ventral lateral PAG (vlPAG), right vlPAG, left lateral PAG (lPAG), and right lPAG, respectively. ROI masks were drawn on the template brain image for all PET data which to be coregistered. These ROI masks were used to extract the AI values from each PET brain image.

The paired brain areas with significantly correlated inter-subject variability of brain FDG consumption (*P* < 0.05, false discovery rate (FDR) corrected) were shown in the matrix with warm and cold color mapping for positive and negative correlation, respectively, and the non-significances were colored with black. For example, if the column x, row y of a matrix refer to the brain areas of A V.S. B, which were significantly and positively correlated in their FDG consumption, it would be marked with warm color, and the brightness of the color refers to the corresponding Pearson’s correlation coefficient r value shown in the color bar.

### Data analysis

We conducted the statistical analysis using SigmaStat (version 3.11) and presented the results as mean ± SD. Data were considered statistically significant at *P* < 0.05. For the behavioral test, we evaluated the differences in withdrawal thresholds using one-way repeated measures ANOVA and Tukey’s post hoc multiple comparison. For mechanical stimulation before FDG-PET scanning, we evaluated the differences in withdrawal ratio among various conditions using one-way repeated measures ANOVA and Tukey’s post hoc multiple comparison. For PET image analysis, we used SPM8 to conduct voxel-based analysis of PET images based on a paired t-test comparison. We considered *P* < 0.05 (uncorrected) to be statistically significant. T-value maps of the results were superimposed on coronal views of a representative MRI images to define voxels, showing significant changes. For ROI analysis, we evaluated the differences in the AI among various conditions using one-way repeated measures ANOVA and Tukey’s post hoc multiple comparison.
